# Changes in Anterior Chamber Depth after Combined Phacovitrectomy

**DOI:** 10.4274/tjo.71601

**Published:** 2016-08-15

**Authors:** Gökhan Gülkılık, Sevil Karaman Erdur, Merve Özbek, Mustafa Özsütçü, Mahmut Odabaşı, Göktuğ Demirci, Mehmet Selim Kocabora, Mustafa Eliaçık

**Affiliations:** 1 İstanbul Medipol University Faculty of Medicine, Department of Ophthalmology, İstanbul, Turkey

**Keywords:** Phacovitrectomy, anterior chamber depth, intraocular lens, postoperative refractive outcomes

## Abstract

**Objectives:**

To evaluate changes in anterior chamber depth (ACD) and postoperative refractive outcomes after combined phacovitrectomy.

**Materials and Methods:**

This study included 10 eyes of 10 patients that underwent combined phacovitrectomy (study group) and 14 eyes of 14 patients that underwent phacoemulsification surgery (control group) at İstanbul Medipol University Ophthalmology Department. Preoperative and 3-month postoperative best corrected visual acuity (BCVA), ACD, change in ACD and refractive outcomes were compared between the two groups.

**Results:**

Preoperative ACD, postoperative ACD at 3 months and change in ACD were similar between two groups (p=0.403, p=0.886, p=0.841). Postoperative mean refractive outcomes were 0.22±0.51 diopter in the phacovitrectomy group and -0.39±0.53 diopter in the phacoemulsification group (p=0.019). BCVA was increased in both groups (p=0.001).

**Conclusion:**

Postoperative refractive outcomes in eyes that underwent combined phacovitrectomy are different from those in eyes that underwent only phacoemulsification surgery. This is important in determining preoperative intraocular lens power before combined phacovitrectomy.

## INTRODUCTION

The incidences of both cataract and vitreoretinal pathologies increase with advancing age. Cataract can interfere with visualization and complicate vitreoretinal surgery. Furthermore, vitreoretinal surgery precipitates or accelerates cataract formation. According to one study, the rate of cataract surgery in phakic patients after vitreoretinal surgery was 75% at 1 year and 90% at 2 years.^1^

Recent improvements in vitrectomy devices and surgical techniques have allowed the successful execution of phacoemulsification combined with pars plana vitrectomy (PPV), which has been termed ‘phacovitrectomy’. Phacovitrectomy has various advantages and disadvantages.^2^ One of the drawbacks of phacovitrectomy is deviations in the refractive outcomes.^3^ The literature contains varied results, but myopic shift has been most commonly reported after phacovitrectomy.^4,5,6^ Errors in axial length measurement, changes in anterior chamber depth (ACD), and aqueous humour replacing the vitreous all affect the refractive index and lead to myopic shift.^3^ ACD determines the effective lens position (ELP), which is an important factor in modern intraocular lens (IOL) power calculation formulas. Every 1 mm change in ELP results in 1.5 diopters (D) of deviation.^3^

The purpose of this study was to evaluate changes in ACD and refractive outcomes after phacovitrectomy.

## MATERIALS AND METHODS

This prospective study included 24 eyes of 24 patients operated on between January and July 2013 at the İstanbul Medipol University Faculty of Medicine, Ophthalmology Clinic. The study was approved by the İstanbul Medipol University Ethics Committee. The study group consisted of 10 eyes of 10 patients that underwent phacovitrectomy; the control group included 14 eyes of 14 patients that underwent phacoemulsification only. The study group included patients with cataract having PPV for various pathologies [epiretinal membrane (ERM) and vitreomacular traction (VMT)] whose axial length could be measured using the IOLMaster (Carl Zeiss Meditec AG, Jena, Germany), while the control group included patients having cataract surgery whose axial length could be measured with the IOLMaster. Patients with previous intraocular surgery, refractive error greater than ±5 D, pseudoexfoliative or traumatic cataract, and patients requiring silicone or gas endotamponade were not included in the study. Patients for whom axial length could not be measured with the IOLMaster were excluded.

All patients underwent a complete ophthalmologic examination and provided informed consent after electing surgery. After measuring axial length with the IOLMaster, the SRK-T formula was used to calculate the IOL power for a target refraction of -0.50 D. Because the IOLMaster does not always provide reliable ACD measurements in pseudophakic eyes, ACD was measured using A-scan ultrasonography (Eye Cubed™, Ellex, Adelaide, Australia).^7^ All measurements were performed by the same technician prior to pupil dilation.

All procedures were performed by the same surgeon (G.G.). In the study group, the vitrectomy procedure followed phacoemulsification and IOL implantation. For all patients, phacoemulsification was performed through a 2.8 mm superior corneal incision and a SN60WF hydrophobic acrylic IOL (Alcon, Foxworth, TX, USA) was implanted in the capsular bag. The vitreoretinal procedure was performed as a standard 23-gauge vitrectomy with membrane peeling. Air, gas or silicone oil tamponade was not used in any of the patients.

Postoperative evaluation was done in the third month. A full ophthalmologic examination including best corrected visual acuity (BCVA), A-scan ultrasonography and ACD measurement were performed. Refractive errors were measured by autorefractometer (Topcon KR 8800, Oakland, NJ, USA) and recorded as spherical equivalent.

BCVA, preoperative and postoperative ACD, change in ACD and refractive errors were compared between the two groups.

The Wilcoxon signed ranks test and Mann-Whitney U test were applied during statistical analysis using SPSS version 15.0 software. The level of significance was accepted as a=0.05.

## RESULTS

Twenty-four eyes of 24 patients were evaluated in this study. The 10 patients in the phacovitrectomy group underwent vitreoretinal surgery due to ERM (7 patients) or VMT syndrome (3 patients). No intraoperative or postoperative complications occured in any of the patients. All patients were followed for the full 3-month follow-up period. The demographic distributions and preoperative ACD, preoperative BCVA, axial length and calculated IOL power are shown in Table 1. The two groups had comparable age and gender distributions, as well as axial length, preoperative ACD and calculated IOL power (p>0.05). The control group had significantly higher preoperative BCVA than the study group (p=0.0001). In the study group, BCVA increased significantly from 0.89±0.12 logMAR preoperatively to 0.38±0.15 logMAR at postoperative 3 months (p=0.001). BCVA of the control group also increased significantly from 0.56±0.17 logMAR preoperatively to 0.50±0.06 logMAR at postoperative 3 months (p=0.001).

The pre- and postoperative ACD values, changes in ACD and spherical equivalents at postoperative 3 months for both groups are shown in Table 2. Mean ACD of the phacovitrectomy group was 2.87±0.26 mm preoperatively and 4.11±0.54 mm at postoperative 3 months. The mean change in ACD was 1.24±0.43 mm, which was a statistically significant increase (p=0.001). In the control group, the mean ACD was 2.91±0.41 mm preoperatively and increased by a mean of 1.27±0.33 mm to reach 4.18±0.39 mm at postoperative 3 months. This was also a significant increase in depth (p=0.001). Preoperative ACD, 3-month postoperative ACD and change in ACD were similar between the two groups (p=0.403, p=0.886, p=0.841, respectively). There was a significant difference in spherical equivalent at postoperative 3 months between the phacovitrectomy group (0.22±0.51 D) and the control group (-0.39±0.53 D) (p=0.019).

## DISCUSSION

Phacovitrectomy is being performed with increasing frequency. Advances in both cataract and vitreoretinal surgery play a role in this trend. Along with higher anatomic success rates, postoperative refractive outcomes have gained importance in combined surgery.^8^ Differing results are reported in the literature, but the most common refractive outcome of phacovitrectomy is myopic shift.^4,5,6^ These studies, most of which use ultrasonic biometry, have demonstrated that errors in the measurement of axial length are usually the source of this myopic shift.^8^Kovács et al.^6^ recommended Ultrasonic biometry measures axial length as the distance between the cornea and the surface of the internal limiting membrane. Axial length may be measured shorter in patients with increased macular thickness, thus causing a deviation toward myopia. Kovács et al.6 recommended using optical coherence tomography (OCT) to measure macular thickness in ERM patients and using this value to correct axial length while doing biometric calculations. Patel et al.^9^ claimed that adjusting the lens strength to be slightly hypermetropic in patients with macular pathology will reduce myopic shift. In optical biometry, axial length is measured as the distance between the cornea and the retinal pigment epithelium, and increased macular thickness can result in underestimation of axial length.^10^ Optical biometry requires patient fixation, which allows for more accurate axial length measurement. However, pathologies such as ERM which cause eccentric fixation may result in the measurement of axial length on a different axis and lead to refractive deviations.^11^

ELP has been demonstrated as one of the factors that influences biometric calculation. ELP is determined by ACD, axial length, corneal thickness and IOL-related factors.^12^ As the actual position is difficult to determine, ELP is based on the ACD measurement. It has been reported that after cataract surgery ACD increased by approximately 1.4 mm and ELP shifted posteriorly.^13^ According to another study, an even larger increase in ACD occured after phacovitrectomy and the IOL tended to be placed more posteriorly.^14^ In contrast, Hamoudi and La Cour^3^ observed that after phacovitrectomy, posterior capsule fibrosis was more severe than in cataract surgery alone, and that this caused the ELP to move anteriorly. Suzuki et al.^5^ found that using intraocular gas tamponade shifted the ELP forward, resulting in a change toward myopia. Schweitzer and Garcia^15^ evaluated the effect of gas tamponade on postoperative refractive values in eyes having phacovitrectomy and reported that the postoperative refraction of eyes that received gas tamponade was -0.30 D, compared to +0.16 in eyes that did not receive gas tamponade.

This study involved the comparison of ACD changes and refractive values 3 months postoperatively in 10 eyes of 10 patients having vitrectomy and 14 eyes of 14 patients having only phacoemulsification. Intraocular tamponade was not used in any of the patients during phacovitrectomy. Both groups showed comparable preoperative and postoperative ACD values and ACD change. There was a significant difference between the groups in refractive values at 3 months postoperatively, with the phacovitrectomy group showing a more hypermetropic shift compared to the control group. These results are consistent with those obtained by Schweitzer et Garcia^15^ in eyes with gas tamponade. Our findings of comparable ACD values but different refractive outcomes between the two groups suggests that either ACD alone does not represent ELP, or that in addition to ELP other factors such as postoperative axial length and macular edema also play a role in biometric calculation. In patients with macular pathology, preoperative axial length measurements may differ from postoperative measurements due to eccentric fixation or changes in macular thickness.^8^ Because the aim of the current study was to evaluate changes in ACD, we did not evaluate changes in axial length. Therefore, the weakest aspect of this study is that macular thickness and postoperative axial length were not measured by OCT. The myopic shift occurring in patients after phacovitrectomy cannot be explained by changes in ACD alone. Preoperative macular thickness appears to be the most important factor in postoperative myopia.

The algorithms in current biometric formulas are specific for phacoemulsification; algorithms for phacovitrectomy have not been developed yet. It has not been clearly established how this affects refractive outcomes. Developing algorithms for phacovitrectomy and using these for biometric calculations may improve the refractive success of phacovitrectomy.

## CONCLUSION

The refractive outcomes of eyes having phacovitrectomy may differ from those of eyes having phacoemulsification alone. Determining the factors that contribute to this difference is essential in order to improve the refractive outcomes of phacovitrectomy.

### Ethics

Ethics Committee Approval: İstanbul Medipol University Ethics Committee 10840098-604.01.01-E.2754, Informed Consent: It was taken.

Peer-review: Externally and internally peer-reviewed.

## Figures and Tables

**Table 1:**
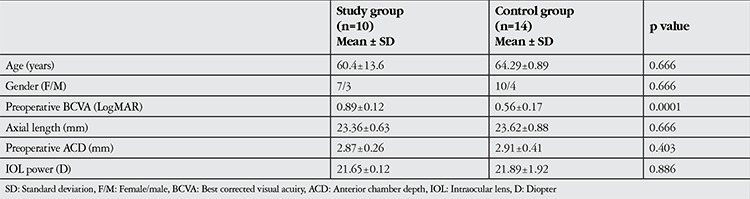
Comparison of demographic and preoperative characteristics between groups

**Table 2:**
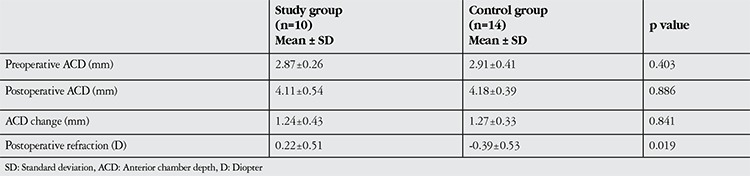
Comparison of postoperative characteristics between groups
